# Effect of Cold-Plasma-Treated Phosphate Solution to Substitute Partial Nitrite on the Color, Texture, and Flavor of Smoked Sausage

**DOI:** 10.3390/bioengineering9120794

**Published:** 2022-12-13

**Authors:** Dejuan Meng, Xinyu Yang, Huan Liu, Dequan Zhang, Chengli Hou, Zhenyu Wang

**Affiliations:** 1 Institute of Food Science and Technology, Chinese Academy of Agricultural Sciences, Beijing 100193, China; 2 Key Laboratory of Agro-Products Processing, Ministry of Agriculture and Rural Affairs, Beijing 100193, China

**Keywords:** cold plasma, nitrite, smoked sausage

## Abstract

There are several alternative technologies to nitrite use in meat products, including cold plasma. In this study, a cold-plasma-treated phosphate solution was added to smoked sausage, as a new ingredient. Subsequently, the color, texture, and flavor of the samples were analyzed. The results showed that, compared with nitrite (0.075 g/kg nitrite added to sausage), the addition of 30~90% nitrite and cold-plasma-treated phosphate solution had no significant effect on the a* value or the relative content of oxygenated myoglobin (*p* > 0.05). The amount of residual nitrite in the smoked sausage prepared with the addition of 30~70% nitrite and cold-plasma-treated phosphate solution was significantly lower than that of the nitrite-treated group. The addition of nitrite combined with cold-plasma-treated phosphate solution had no significant effects on the texture (hardness, springiness, cohesiveness, and resilience) or the sensory evaluation of the smoked sausage. A total of 69 volatile compounds were detected, and 20 of them had VIP (Variable Importance Plot) scores higher than one. In conclusion, cold plasma treatment represents a potential technology to partially substitute nitrite. This study provides new methods for the application of this nitrite substitute.

## 1. Introduction

Smoked sausage is a famous meat product in China [1,2], marked by its ivory color, rich nutrition, and unique flavor, and it is highly favored by citizens in Yunnan, Guizhou, Sichuan, and other provinces. The traditional production of sausage involves selecting pig foreleg meat and back fat; adding salt, sugar, liquor, and other seasonings; and then pickling, filling, and smoking. Nitrite is usually added to the smoked sausage, to improve the color, flavor, and anti-oxidation and antibacterial properties [3,4,5], but nitrite represents a potential hazard to humans, due to NO_2_^-^ being directly associated with gastrointestinal tumors, stomach cancers, and certain diseases. In the human body, a concentration of 33–250 mg of nitrite per kilogram of weight is fatal, while a concentration of 0.4 to 200 mg of nitrite per kilogram of body weight is enough to cause methemoglobinemia [6,7,8]. Therefore, exploring nitrite replacement technologies is a common challenge in the meat industry.

The replacement technologies for nitrite mainly include the following three categories: (1) Natural plant components, including celery powder, cherry powder, beetroot, and radish powders, etc. Viktorija Eisinaitė [9] studied freeze-dried celery as a substitution for nitrate in cold smoked sausages, which could improve the color of the product, but had the disadvantage of being used with starter cultures, in order to successfully harness the fermentation and ripening processes. Maristela Midori Ozaki et al. [10] reported that the addition of 1% radish powder could be the best choice for nitrite substitution. (2) Microbial fermentation substance replacement, including *Staphylococcus*, *Nisin*, etc.; Li et al. [11] found a potential solution for nitrite substitution in meat products and also demonstrated that the a* values of *S. xylosus* sausages were almost the same as those for meat with nitrite added in raw meat batters. (3) Chemical additive replacement, including antioxidants, etc. Chiara Aquilani [12] demonstrated that natural antioxidants, as a substitution for nitrite, did not influence the overall acceptability and other physicochemical indexes of products. All of these technologies have some limitations, such as typical flavor defects or color flaws.

As an emerging non-thermal food processing technology, cold plasma (CP) technology has attracted the interest of many researchers and scientists around the world [13]. Initially, CP was applied to improve the printing and adhesion properties of polymers, enhancing the surface energy of materials and the diversity of usage domains in electronics. It has been applied in glass processing, as well as in production of paper and other products [14]. New research has indicated that CP could be a powerful technology for the food industry. The technology offers great advantages in terms of microbial decontamination. The results of different studies on fat oxidation were inconsistent. Wang et al. [15] found that a dielectric barrier discharge cold plasma treatment could significantly improve the color and myoglobin contents, while accelerating the lipid oxidation and spoilage and deterioration of beef during storage. Chen [16] showed that, compared with a control group and one with added nitrite, plasma treatment for 15–45 min increased the redness of a sample by 9.30~31.40%, with plasma treatment inhibition increasing the lipid oxidation values from 86.69% to 89.89%. Zhang found that a dielectric barrier discharge cold plasma treatment for meat could significantly decrease the redness and pH [17]. Jung found that cold plasma was a safe technology and could be used in meat processing [18].

Based on a previous study [16], direct plasma treatment could enhance the redness values of roast meat samples, and the overall sensory score with plasma treatment for 30 min was higher than that of the control treatment samples, and similar to the nitrite treatment group. Therefore, in this study, a cold-plasma-treated phosphate solution and different concentrations of nitrite were applied, to analyze the effect of cold-plasma-treated phosphate solution on the color, texture, and flavor of smoked sausage. This study provides a new concept for the application of cold plasma technology to replace nitrite in the meat industry.

## 2. Materials and Methods

### 2.1. Reagents and Materials

Apple wood (Botou Runsheng Wood Products Co., Ltd., Tianjin, China); edible salt (Beijing Salt Co., Ltd., Beijing, China); MSG (Meihua Biotechnology Group Co., Ltd., Langfang, China); white sugar (Beijing Sugar Tobacco and Tobacco Group Co., Ltd., Beijing, China); white pepper (Zhumadian Flavoring Group Co., Ltd., Zhumadian, China); liquor (Beijing Shunxin Agriculture Co., Ltd., Beijing, China); and pepper powder (Beijing Bond Flavoring Co., Ltd., Beijing, China) were all used in this experiment.

### 2.2. Smoked Sausage Manufacture

First, 100 mL of phosphate solution was placed into a 500 mL tapered flask and then placed under the spray head of the cold plasma equipment (SY-JXDW-01, Suzhou Fengyuanbao Agricultural Science and Technology Co., Ltd., Suzhou, China). The cold plasma discharge reached the surface of the liquid. The parameters were as follows: 40 W power, 30 L/min flow rate, and 45 min. Finally, the treated solution was stored in a 100 mL centrifuge tube, before being placed in a −20 °C refrigerator.

Samples from 12 pigs (Duroc–Landrace–Yorkshire, 6 months old, 75 kg) were purchased from Beijing Shunxin Agriculture Co., Ltd.). Next, the 12 boars (divided into 3 batches and 3 repeats, with 4 pigs in each batch) were slaughtered after 12 h at 4 °C, and the pigs’ front leg meat and back fat were taken. The raw meat (anterior tip meat and dorsal fat) was removed from the fascia, fat, and other trimmings, and a grinding mechanism containing a 5 mm plate was used to produce minced meat. The ground raw meat was weighed separately, with a 7:3 fat-to-lean mass radio, and was fully mixed. The ingredients (27 g/kg of salt, 1 g/kg of sugar, 1 g/kg of liquor, 4 g/kg of pepper powder, 0.5 g/kg of thirteen spice, and 1.5 g/kg of glutamate) were mixed evenly into the raw meat, the cold-plasma-treated phosphate solution was added, stirred evenly, and filled into a 30–32 mm diameter pig casing. Each smoked sausage was about 15 cm long, After knotting, they were then dried in a constant temperature humidity box (the specific drying parameters were: 40 °C, 15 min→55 °C, 5 h→40 °C, 2 h). After drying, apple wood was used as a smoking wood for 3 min at 60 °C, and vacuum packaging was adopted after natural cooling (Table 1).

### 2.3. Instrumental Color Evaluation

The smoked sausage was cooled to room temperature (26 ± 1 °C), then the color was determined using a CM-600D Minolta colorimeter (Konica Minolta Sensing Americas Inc., Ramsey, NJ, USA) with a CIE D65 10°standard observer. The specific method involved cutting 1 cm thick slices from the front, middle, and back end of the smoked sausage; vertically determining the L*, a*, and b* values of the different parts, with parallel measurements 3 times; and calculating the average value.

The myoglobin percentage of smoked sausages was measured using CM-600D Minolta colorimeter, with some modifications, according to Li [19] and Wang et al. [20]. The reflectance values from 360 to 740 nm were recorded with a Minolta instrument at 10 nm intervals. The reflectance values at specific wavelengths (503, 525, 557, and 582 nm) were calculated using linear interpolation. The percentages of deoxy myoglobin (DMb), oxymyoglobin (OMb), and metmyoglobin (MMb) were determined using the method of Zhao et al. DMb = −0.534R_1_ + 1.594R_2_ + 0.552R_3_ − 1.329, OMb = 0.772R_1_ − 1.432R_2_ − 1.659R_3_ + 2.559, MMb = −0.159R_1_ − 0.085R_2_ + 1.262R_3_ − 0.520, Where R_1_ = A_582_/A_525_, R_2_ = A_557_/A_525_, R_3_ = A_503_/A_525_.

### 2.4. Texture Profile Analysis (TPA)

The TPA of the smoked sausages was assessed using the method proposed by Zhu et al., with some modifications [21]. The sausage sample was cut into a 20 mm diameter and 15 mm high block, the cube was placed in the center of the worktable in a texture analyzer (TA-XT plus, Stable Micro Systems Co., Ltd., London, UK). The sample was compressed to 50% of the original height at room temperature using a P/50 probe with a double compression cycle. The parameters were set as follows: a trigger force of 5 g, a pre-test speed of 2 mm/s, a test speed of 1 mm/s, and a post-test of speed 3 mm/s. The hardness, springiness, cohesiveness, and resilience were obtained using different software. The TPA attributes of each smoked sausage were averaged from two measurements, the tissue was used to determine the TPA, each sample test was performed 6 times, and the average value was taken.

### 2.5. Analysis of Residual Nitrite

The residual nitrite in the eight groups of smoked sausages was determined using a nitrite detector (CSY-SY, FenXi Instruments Manufacturing Co., Shenzhen, China), based on the hydrochloride naphthylenediamine method described by AOAC.

### 2.6. Analysis of Volatile Compounds

#### 2.6.1. E-Nose Analysis

E-nose analysis of the sausages was performed using the method proposed by Li et al. [22], First, after each treatment, 2.0 g of smoked sausage was placed in a 20 mL flavor bottle and sealed, then begin left to stand at 25 ± 1 °C for 30 min. The volatile odor of the samples was analyzed using an E-nose. The E-nose collection time and cleaning time were 60 s and 180 s, respectively, and the sample gas flow rate and cleaning gas flow rate were 300 mL/min and 600 mL/min, respectively. The response signal from 56 to 58 s was taken as the response value of the sample. E-nose analysis used an Odour Sensing System E-nose (PEN 3.5, Airsense, Schwerin, Germany). The response type of each sensor to the different substances are shown in Table 2.

#### 2.6.2. GC-IMS Analysis

To clarify the effect of the plasma treatment on the flavor of the product, GC-IMS was used to determine the flavor substances of the smoked sausages. Volatile compounds in the smoked sausages were analyzed using gas chromatography ion mobility spectroscopy (GC-IMS), in line with the method proposed by Liu et al. [23].Then, a 2.0 g smoked sausage sample was directly transferred into a 20 mL headspace vial, incubated at 60 °C for 20 min, and directly injected into an IMS commercial instrument (Flavor Spec^®^) from Gesellschaft für Analytische Sensorsysteme mbH (Dortmund, Germany). Triplicate injections and sample analyses were performed. Chromatographic separation was performed on an MXT-WAX capillary column (30 m × 0.53 mm, 1 μm film thickness) maintained at 60 °C. The incoming sample intake volume was 500 μL. Nitrogen gas was employed as a carrier gas with the flow ramp starting at 2 mL/min for 2 min, then this was increased to 10 mL/min after 10 min, to 100 mL/min after 20 min, and finally to 100 mL/min after 25 min. The total GC runtime was 45 min. VOCal software was used to view the spectrogram and for qualitative quantification of the data. Spectral map differences between samples were directly compared using the Reporter and Gallery Plot plugin. Both sets of software were provided by G.A.S. (Dortmund, Germany).

### 2.7. Sensory Analysis

Sensory evaluation was undertaken by a panel of 10 semi-trained tasters, following the protocols of Kehlet et al. [24] and Song et al. [25]. The panel consisted of 10 researchers from the Meat Science and Nutrition Engineering Innovation Team (50% male/female, age range 20 to 40 years). Prior to the evaluation stage, the sensory characteristics of the smoked sausages (color, texture, flavor, etc.) were evaluated, and sensory evaluation methods (the introduction of scoring principles to the evaluator) were used. Each sample was numbered with a three-digit random number and randomly provided to the taster. The tasters tasted and scored each treatment three times. Each slice (with a 1 cm thickness) was cut and presented to each member of the sensual assessment personnel. Panelists rinsed their mouths with water and ate some bland crackers between samples. The sausage color (1 = not red at all, 10 = extremely red), smoked flavor (1 = extremely undesirable, 10 = extremely desirable), juiciness (1 = extremely dry, 10 = extremely juicy), chewing (1 = extremely undesirable, 10 = extremely desirable) and overall evaluation (1 = extremely undesirable, 10 = extremely desirable) were evaluated using ten-point scales.

### 2.8. Statistical Analysis

The results were expressed as the mean ± standard deviation. All results were analyzed using one-way ANOVA, followed by least significant differences (LSD) multiple comparison tests (*p* < 0.05), using the SPSS statistical software (SPSS Ver. 17, SPSS Inc., Chicago, IL, USA). Partial least squares discrimination analysis (PLS-DA) was used to analyze the flavor and sensory scores of the 8 groups of smoked sausages using SIMCA software (version 14.1, Sartorius AG, Gottingen, Germany).

## 3. Results

### 3.1. Color of Smoked Sausage

The L*, a*, and b* values of smoked sausages in the different treatment groups are shown in Table 3. Compared with the control treatment group, the smoked sausages prepared by adding only 75 mg/kg of sodium nitrite showed a significant decrease in L* and b*, and the a* values were significantly higher (*p* > 0.05). Compared with the control treatment group, the a* value of the smoked sausage prepared using the phosphate solution with cold plasma increased by 1.65–2.49. Compared with the nitrite-treated group (75 mg/kg), the 30–90% nitrite group was used combined with the phosphate solution treated with cold plasma, and there was no significant difference in the redness of the smoked sausage.

**Figure 1:**
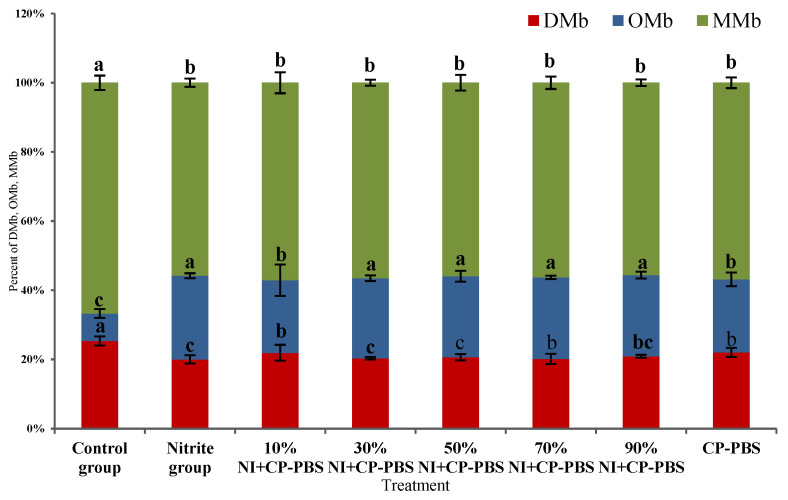
Effect of different treatments on the relative contents of the three myoglobins in smoked sausage. ^a~c^ Different letters indicated significant differences among the treatment groups at *p* <0.05.

The relative content of the three myoglobins of the smoked sausage showed in Figure 1, that there was no significant difference (*p* > 0.05) compared with 30% to 90% sodium nitrite and the nitrite-treated group (75 mg/kg). The relative content of the three myoglobins in the smoked sausage smoked sausages prepared with the cold-plasma-treated phosphate solution was lower than that of the nitrite-treated group.

### 3.2. Texture of the Smoked Sausages

Results from the analysis of the texture of the eight groups of smoked sausage are shown in Table 4. Compared with the nitrite-treated group (75 mg/kg), the hardness value of the smoked sausage treated with only plasma was 39.61% lower and had no significant difference in springiness, cohesiveness, or resilience. However, there was no significant difference in the texture of the smoked sausages prepared with the use of 10–90% nitrite and the phosphate solution treated with cold plasma.

### 3.3. Residual Nitrite in the Smoked Sausage

**Figure 2:**
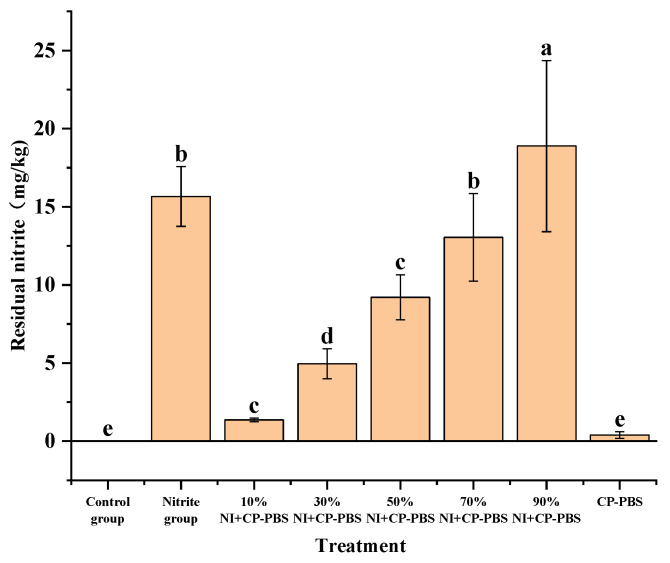
Effect of different treatments on residual nitrite in the smoked sausages. ^a~e^ Mean values in the same row followed by different letters are significantly different (*p* < 0.05).

The residual nitrite levels in the smoked sausages in the eight groups are shown in Figure 2. When the cold-plasma-treated phosphate solution was added, and as the nitrite content was increased, the nitrite residues in the smoked sausage also increased. Compared with the nitrite-treated group (75 mg/kg), no significant differences in nitrite residues were found in smoked sausages prepared by adding 70% sodium nitrite and the cold-plasma-treated phosphate solution. Moreover, the nitrite residues in smoked sausages prepared by adding 90% sodium nitrite with cold-plasma-treated phosphate solution were 20.55% higher than in the nitrite-treated group. The residual nitrite in the smoked sausages was significantly lower in all other treatment groups than in the nitrite-treated group (*p* < 0.05).

### 3.4. Volatile Compounds

#### 3.4.1. Electronic Nose Analysis

**Figure 3:**
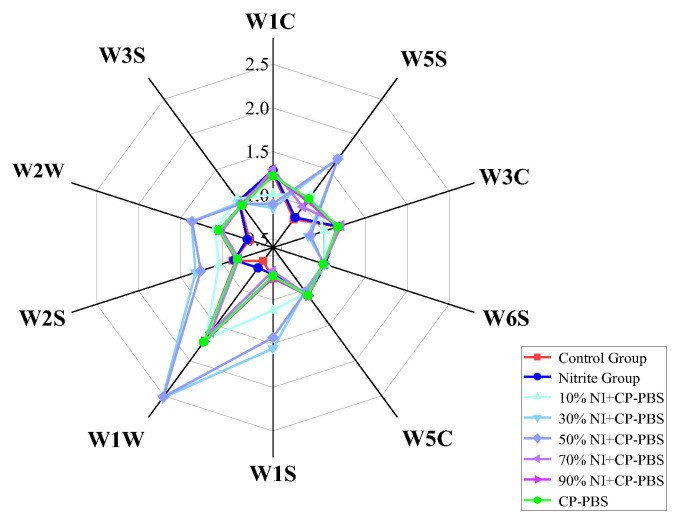
Radar of the volatile flavor substances of smoked sausage in different treatment groups.

An electronic nose was used to quickly identify the smoked sausages in different treatment groups. As seen in Figure 3, the response signals of the different treatment groups to the electronic nose were different. Compared with the control group, the response signal for the W3S sensor was higher in the nitrite group than in the control group, while the response values of the remaining sensors did not show any significant changes (*p* > 0.05), indicating that the addition of nitrite could increase the methane-aliph content in the samples. Compared with the nitrite group, the response values for the W1C, W3C, and W5C sensors were significantly lower, and the response values for the W5S, W1S, and W2S sensors were significantly higher than those for the nitrite-treated group when 10% to 50% nitrite was added in combination with the cold-plasma-treated solution; the response signals for the W6S sensor were significantly lower for samples prepared with 50% to 90% nitrite with the plasma-treated phosphate solution, and the samples prepared with cold-plasma-treated phosphate solution were significantly lower than for the nitrite-treated group. The addition of cold-plasma-treated phosphate solution increased the response of the samples to W1W and W2W, as compared with the nitrite-treated group; and for the W3S sensor, the addition of 30% to 90% sodium nitrite and cold-plasma-treated phosphate solution decreased the response.

#### 3.4.2. Analysis of Gas Phase Ion Migration Spectrum of Smoked Sausage Flavor Substances in the Different Treatment Groups

**Figure 4:**
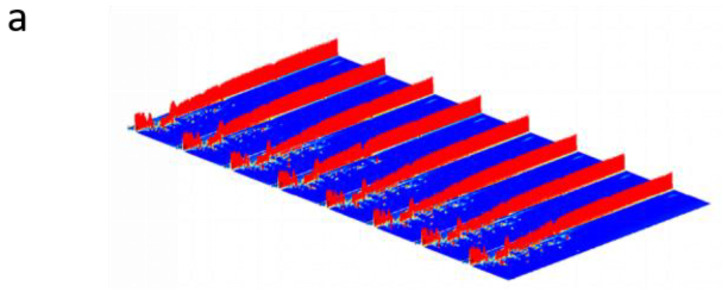

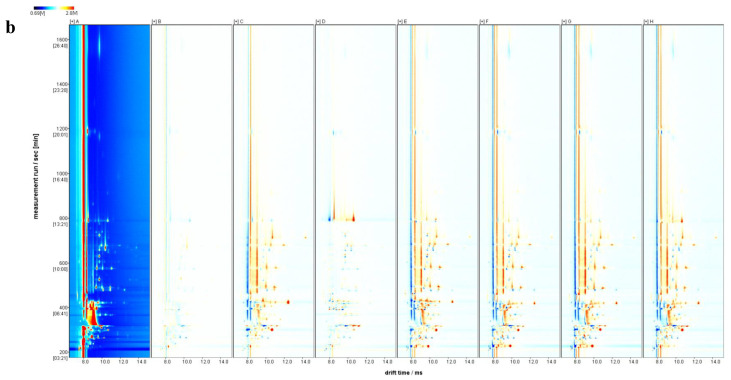
GC-IMS 3D profiles (**a**) and 2D profiles of the volatile flavor substances of the smoked sausages in the different treatment groups (**b**).

Figure 4a provides a GC-IMS 3D representation of the volatile compounds of the different treatment groups, according to the LAV analysis, and Figure 4b is a top view. This figure visually shows that the volatile compound species varied significantly among the treatments. The Y-axis is the retention time of the GC, and the X-axis is the ion relative drift time. The red line parallel to the Y-axis indicates the reactive ion peak (RIP) at the X-axis on a 1.0 scale. In the figure, each data point represents a volatile compound, whose intensity information is represented by a color (white represents a low volatile compound concentration, and red represents a higher concentration).

In order to compare the differences with greater precision, a different contrast mode was used. For this, a sample spectrum was collected as a reference, and the other seven samples were successively deducted from the reference. If the volatile compounds content was higher than the reference, the substance turned red, but if the volatile compounds content was lower than the reference, the substance turned blue. Figure 4b shows a 2D map of the IMS for the smoked sausage across the seven treatment groups.

**Figure 5:**
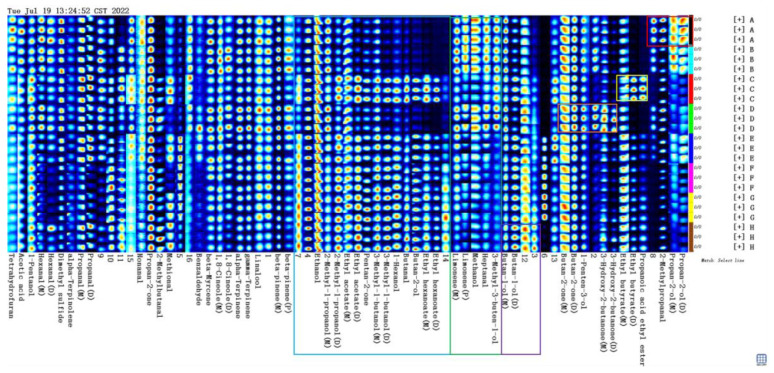
Global overview of the spots identified in the smoked sausage samples in the different treatment groups.

Figure 5 shows the fingerprint of the volatile compounds of the smoked sausage in the different treatment groups. Each row represents all the signal peaks selected in one smoked sausage sample, and each column represents the signal peaks of the same volatile organic compounds in different smoked sausage samples. The full VOC information for each sample and the differences between the samples can be seen in Figure 5. The substances in the red box in Figure 5 were the highest in A, including isopropanol and isobutyral. Volatile compounds in the yellow box in Figure 5 had the highest content in C, including ethyl propionate and ethyl butyrate. The substances in the orange box in Figure 5 had the highest content in D, including 3-hydroxyl-2-butanone, 1-pentene-3-alcohol, and 2-butanone, amongst others. The substance in the purple box in Figure 5 was highest in H, including 1-butanol and others. The substances in the green box in Figure 5 were higher in A, B, and D, including 3-methyl-3-butene-1-alcohol, heptanaldehyde, methanol, and limonene. Some unknown flavor compounds require further characterization.

**Figure 6:**
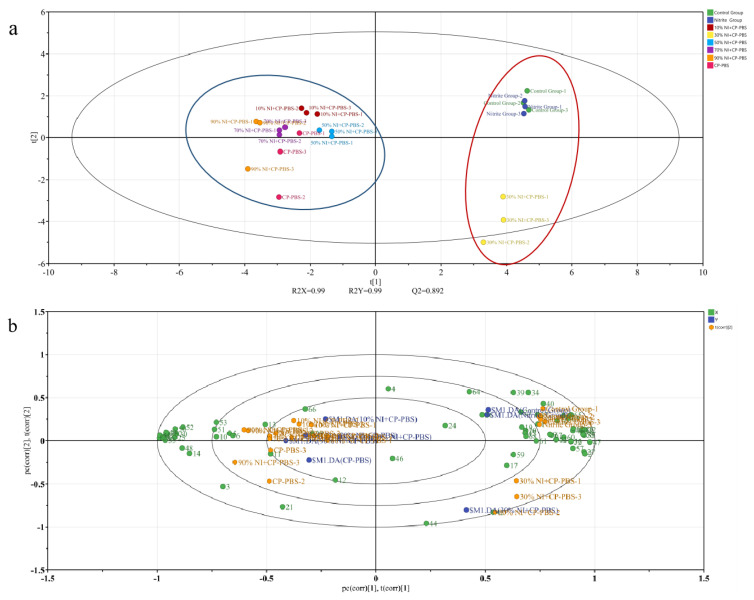

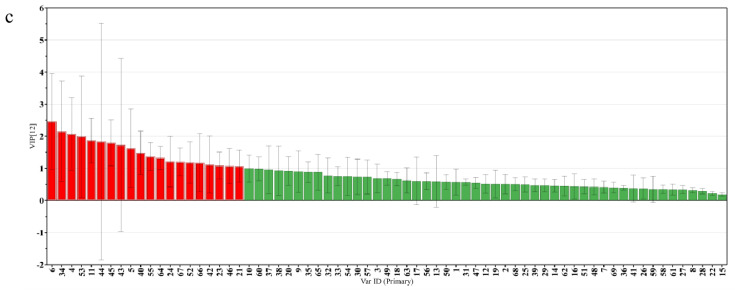
PLS−DA analysis of the volatile compounds during oral processing in the smoked sausage. (**a**) Permutation plot; (**b**) Biplot; (**c**) VIP score.

The addition of cold-plasma-treated phosphate solution decreased the content of Ethyl hexanoate compared to the nitrite-treated group, and the content of Ethyl hexanoate increased with the increase in the nitrite content, following the addition of the same content of cold-plasma-treated phosphate solution. Meanwhile, the addition of cold-plasma-treated phosphate solution increased the content of 2-Methylbutanal compared to the nitrite-treated group. The addition of cold-plasma-treated phosphate solution increased the content of 2-Methylbutanal compared with the nitrite-treated group. As the nitrite was content increased, its content also increased, and the phosphate solution with the addition of cold-plasma-treated had a lower methanol level compared to the nitrite-treated group, which decreased with the increase in nitrite content. PLS-DA analysis was conducted on the flavor substances of the smoked sausages from the different treatment groups, with the smoked sausages from different treatment groups representing the response variable Y. The validity of the supervised model was further verified using a permutation test. Figure 6a shows the results of the PLS-DA permutation test with *p* < 0.05 for the predictive ability, indicating that the model was stable and had a good predictive ability, without overfitting. The red circles mean that the three groups of smoked sausages had similar flavor characteristics, because they are all located at the positive end of the X-axis. The blue circles mean that the five groups of smoked sausages had similar flavor profiles, because they are all located at the negative end of the X-axis. A biplot was used to visually reflect the contribution of each variable on the score plot, as shown in Figure 6b, and a VIP plot was used to screen the key flavor substances of the smoked sausages in the different treatment groups, as shown in Figure 6c; illustrating that 20 volatile compounds with VIP >1 played an important role in the discrimination, namely ethyl acetate, 3-hydroxy-2-butanone, 2-methyl ketone, ethanol, propylene glycol, ethyl butyrate, 8-eudesmol, acetic acid, propionaldehyde, linalool, 2-methylbutyraldehyde, butan-2-one, and butan-1-ol. This shows that these 20 flavor substances were important contributors to the flavor of the smoked sausages.

* In Figure 6b,c, the numbers 1 to 69 represent the 69 volatile flavor substances: 1–16: unidentified substances, 17: Benzaldehyde, 18: Hexanal (M), 19: Hexanal (D), 19: 2-Methyl propanal, 20: 2-Methyl butanal, 21: Heptanal Propanal (M), 22: Heptanal, 23: Propanal (M), 24: Propanal (D), 25: Butanal, 26: Nonanal, 27: 1-Penten-3-ol, 28: 1-Hexanol, 29: 1-Pentanol, 30: 3-Methyl-1-butanol(M), 31: 3-Methyl-1-butanol (D), 32: 2-Methyl-1-propanol (M), 33: 2-Methyl-1-propanol (D), 34: Ethanol, 35: Methanol, 36: Butan-2-ol, 37: Butan-1-ol (M), 38: Butan-1-ol (D), 39: Propan-2-ol (M), 40: Propan-2-ol (D), 41: 3-Methyl-3-buten-1-ol, 42: Linalool, 43: 3-Hydroxy-2-butan one (M), 44: 3-Hydroxy-2-butanone (D), 45: Propan-2-one, 46: Butan-2-one (M), 47: Butan-2-one (D), 48: Pentan-2-one, 49: Ethyl hexanoate (M), 50: Ethyl hexanoate (D), 51: Propanoic acid ethyl ester, 52: Ethyl butyrate (M), 53: Ethyl butyrate (D), 54: Ethyl acetate (M), 55: Ethyl acetate (D), 56: gamma-Terpinene, 57: alpha-Terpinene, 58: beta-Myrcene, 59: alpha-Terpinolene, 60: beta-pinene (M), 61:beta-pinene (P), 62: Limonene (M), 63: Limonene (P), 64: Acetic acid, 65: Dimethyl sulfide, 66: Methional, 67: 1,8-Cineole (D), 68: 1,8-Cineole (D), 69: Tetrahydrofuran.

### 3.5. Effect of Adding Cold Plasma Treated Phosphate Solution on the Sensory Quality of Smoked Sausage

**Figure 7:**
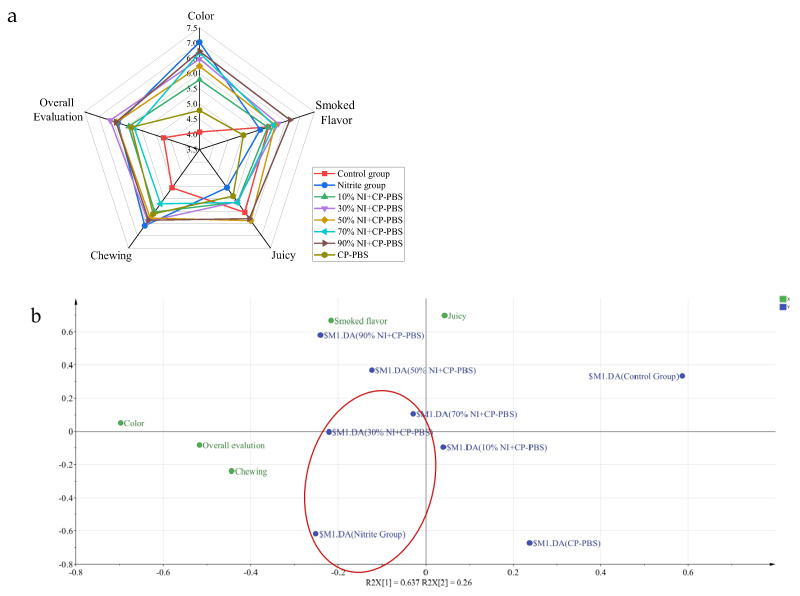
Radar (**a**) displaying the sensory scores of the smoked sausages, color, smoked flavor, juiciness, chewiness, overall evaluation, and PLS−DA bioplot (**b**) analysis of the sensory assessment of the smoked sausage in the different treatment groups.

The color, smoke flavor, juiciness, chewiness, and overall acceptability of the eight different treated smoked sausages are shown in Figure 7, The cold-plasma-treated phosphate solution enhanced the color of the smoked sausage, but for the smoked sausage’s smoke flavor, chewiness, juiciness, and overall evaluation there were no significant differences. Moreover, the cold-plasma-treated phosphate solution had no adverse effects on the sensory quality of the smoked sausage. According to the PLS-DA analysis, the overall sensory quality of the smoked sausages in the 30% sodium nitrite and cold-plasma-treated groups was the closest to that of the nitrite group, indicating that the sensory quality of the smoked sausages in both groups was similar.

### 3.6. Correlation Analysis

**Figure 8:**
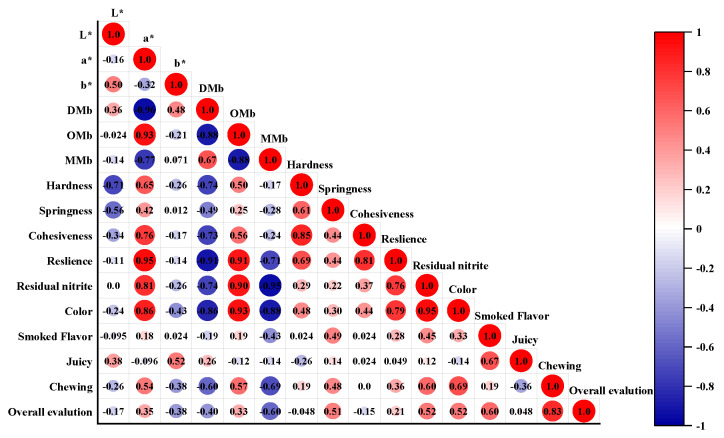
Correlation analysis of the color, texture, residual nitrite, and sensory quality in the smoked sausages.

A correlation analysis of the indicators for the eight treatment groups of smoked sausages was carried out, and the results are shown in Figure 8. The redness of the smoked sausages correlated strongly with the oxygenated myoglobin content, the residual nitrite, and the color in the sensory evaluation. A positive correlation between the texture profile of the smoked sausages was also found. There was also a positive correlation between the nitrite residues in the smoked sausages and the color, smoked flavor, chewiness, and overall acceptability in the sensory evaluation, indicating that residual nitrite is an important factor affecting the edible quality and sensory quality of smoked sausage.

## 4. Discussion

Cold plasma is widely used in the food industry as a non-thermal technology, specifically for microbial decontamination [26,27,28], including of *sporulating* and *spoilage/pathogenic organisms*. In order to avoid adverse effects on the food matrix, researchers often use indirect treatment with cold-plasma-treated solutions, including the treatment of water [29,30], milk [31], sodium pyrophosphate [18], plant protein preparation solutions [32], and other media.

Color is one of the most important indicators that influence consumers when buying meat products. The color changes in smoked sausages with different treatments, this study determined that plasma-treated phosphate solution could enhanced the a* value of the smoked sausages, which was in agreement with Chen’s [15] study, where compared with a control treatment group, cold plasma treatment could significantly improve the a* value of roasted meat samples. Moreover, in the results obtained from Jung’s study [33], it was found that the a* value of cooked meat batter gradually increased with plasma treatment time, when investigating the effect of direct atmospheric pressure cold plasma on the color of minced meat during mixing. This analysis was conducted to investigate how oxygen and nitrogen in the air might be excited by plasma, to produce reactive nitrogen and reactive oxygen, and revealing that the reactive nitrogen produced nitrogen oxides, such as NO, NO_2_, N_2_O_3_ etc. NO_2_, N_2_O_3_, and N_2_O_4_, which could react irreversibly with water to produce nitrite. Nitrite can react with myoglobin in meat to form nitro-myoglobin, producing the red color preferred by consumers. Therefore, this technology can improve the redness of smoked sausages.

Texture is a key factor in determining consumer preference, usually associated with sensory attributes. The texture profile of sausages depends on the matrix structure formed by proteins, water, and the addition of non-meat ingredients [34]. In this study, the value of hardness in the CP-PBS treatment group was significantly lower than in the nitrite group (*p* < 0.05). Cold plasma treatment might also have affected the binding properties of the proteins in used solutions, due to the interactions of the plasma reactive species with amino acids [35]. Compared with the nitrite group, there was no significant effect on the springiness, cohesiveness, and resilience. This is the same result as in MonikaMarcinkowska-Lesiak’s study. Mahnot et al. [34] found no significant effect on the texture of fresh-cut carrots at different times (1, 2, 3, 4, and 5 min) of packaging with cold plasma. It is likely that the amount of added cold-plasma-treated phosphate solution was not sufficient to contribute an appropriate protein network structure during the heating process.

Residual nitrite is an important indicator for ensuring safety. Plasma treatments can produce nitrite, as confirmed by Jung et al. [33] and Faria et al. [36]. Inguglia et al. [37] studied plasma-activated brine as a nitrite source for the curing of beef jerky and showed that, compared with N_2_ gas, air-plasma had resulted in higher nitrite in beef jerky. Jung et al. found that the nitrite content in meat batter gradually increased when increasing the plasma treatment time. This was because the active nitrogen produced by the plasma treatment reacted with H_2_O to produced nitric and nitrous acids, then chemical decomposition produced NO_3_^−^ and NO_2_^−^, thereby increasing the nitrite content of the product. The results showed that plasma technology, as a safe and non-toxic technology, can be an effective alternative to nitrite, using phosphate solutions with plasma.

Flavor is an important part of the sensory attributes [38]. Flavor substances are produced through the Maillard reaction between sugars and amino acids, as well as the heat degradation of lipids and thiamin [39]. Cold plasma can change the content of the volatile compounds of products. The addition of cold plasma treated phosphate solution increased the content of ketones and esters in smoked sausages compared to the nitrite treatment group. The ketones included Butan-2-one, Propan-2-one, 3-Hydroxy-2-butan-one, and Pentan-2-one. Oxidative degradation of fats, oxidation of alcohols, or degradation of esters are the main sources of ketones, and ketones have a low olfactory threshold and contribute to the overall aroma of meat [40]. Ketones are formed from the breakdown of alkoxy radicals and the degradation of amino acids [41]. Esters are important flavor substances in the processing of meat products and play an important role in masking unpleasant odors [42]. Esters with significant differences included ethyl hexanoate, propanoic acid ethyl ester, ethyl butyrate, and ethyl acetate. The formation of esters usually requires a complex chain of reactions, as well as esterification of free fatty acids from fat hydrolysis with alcohols from fat oxidation to form esters [39]. Ke et al. [43] found that cold-plasma-treated air can promote the formation of volatile compounds in dry-cured black carp, such as 1-octene-3-ol, 3-methylbutanal, etc.

In summary, cold plasma improved the smoked sausage color, and the improved qualitative properties could be very beneficial. Although there were material differences in the flavor, we hope to further explore the unrecognized substances and their adverse effects on sensory quality. Therefore, the cold-plasma-treatment phosphate solution could be used to reduce the addition of sodium nitrite and ensure the safety of humans.

## 5. Conclusions

A cold-plasma-treated phosphate solution, combined with 30–70% sodium nitrite, had no adverse effects on the texture, volatile compounds, and sensory evaluation in comparison with smoked sausage prepared with the addition of sodium nitrite. The cold-plasma treatment may represent a potential technology to partially substitute the addition of nitrite in meat production. The mechanisms of the plasma-induced changes in product color and flavor will be further investigated.

## Figures and Tables

**Table 1:** Experimental treatment.

Treatment Groups ^1^	Sodium Nitrite/(mg/kg)	0.067 mol/L Phosphate Solution/(g/kg)	
		CP ^2^	N-CP ^3^
Control group	-	-	150.0
Nitrite group	75.0	-	150.0
10% NI + CP-PBS	7.5	150.0	-
30% NI + CP-PBS	22.5	150.0	-
50% NI + CP-PBS	37.5	150.0	-
70% NI + CP-PBS	52.5	150.0	-
90% NI + CP-PBS	67.5	150.0	-
CP-PBS	-	150.0	-

^1^ Treatment Groups: Control group: without sodium nitrite and plasma treatment; Nitrite group: without plasma treatment and 75 mg/kg sodium nitrite added (by meat weight); 10% NI + CP-PBS: with plasma treatment and 10% sodium nitrite added; 30% NI + CP-PBS: with plasma treatment and 30% sodium nitrite added; 50% NI + CP-PBS: with plasma treatment and 50% sodium nitrite added; 70% NI + CP-PBS: with plasma treatment and 70% sodium nitrite added; 90% NI + CP-PBS: with plasma treatment and 90% sodium nitrite added; CP-PBS: plasma treatment only; ^2^ CP: with plasma treatment; ^3^ N-CP: without plasma treatment.

**Table 2:** Performance of the electric nose sensors.

Name	Sensor Performance
W1C	aromatic
W5S	broad range
W3C	aromatic
W6S	hydrogen
W5C	arom-aliph
W1S	broad-methane
W1W	sulphur-organic
W2S	broad-alcohol
W2W	sulph-chlor
W3S	methane-aliph

**Table 3:** Effect of different treatments on the color of smoked sausage.

	L*	a*	b*
Control group	57.07 ± 0.38 ^a^	5.64 ± 0.52 ^d^	14.18 ± 0.62 ^a^
Nitrite group	54.00 ± 1.60 ^b^	9.63 ± 0.41 ^a^	11.58 ± 0.99 ^bc^
10% NI + CP-PBS	54.22 ± 0.93 ^b^	7.96 ± 0.18 ^bc^	10.97 ± 0.43 ^c^
30% NI + CP-PBS	53.53 ± 3.07 ^b^	8.81 ± 1.02 ^abc^	11.16 ± 0.94 ^c^
50% NI + CP-PBS	55.36 ± 2.18 ^ab^	8.83 ± 1.63 ^abc^	12.71 ± 0.52 ^b^
70% NI + CP-PBS	54.52 ± 1.90 ^b^	9.16 ± 1.20 ^ab^	11.62 ± 0.79 ^bc^
90% NI + CP-PBS	54.66 ± 0.83 ^ab^	8.81 ± 1.42 ^abc^	12.01 ± 1.72 ^bc^
CP-PBS	55.47 ± 1.41 ^ab^	7.71 ± 0.10 ^c^	12.31 ± 1.03 ^bc^

^a~c^ Mean values in the same row followed by different letters are significantly different (*p* < 0.05).

**Table 4:** Effect of different treatments on the texture of smoked sausages.

Treatment	Hardness/g	Springiness	Cohesiveness	Resilience
Control group	8171.58 ± 2333.24 ^ab^	0.80 ± 0.09 ^a^	0.29 ± 0.08 ^ab^	0.09 ± 0.03 ^ab^
Nitrite group	10388.44 ± 1742.99 ^a^	0.81 ± 0.05 ^a^	0.33 ± 0.07 ^ab^	0.12 ± 0.03 ^ab^
10% NI + CP-PBS	7768.63 ± 1682.04 ^ab^	0.76 ± 0.12 ^a^	0.29 ± 0.04 ^ab^	0.09 ± 0.01 ^ab^
30% NI + CP-PBS	8363.80 ± 2935.30 ^ab^	0.88 ± 0.13 ^a^	0.29 ± 0.09 ^ab^	0.10 ± 0.04 ^ab^
50% NI + CP-PBS	8070.14 ± 2889.10 ^ab^	0.82 ± 0.27 ^a^	0.31 ± 0.10 ^ab^	0.11 ± 0.05 ^ab^
70% NI + CP-PBS	9923.35 ± 3471.28 ^ab^	0.79 ± 0.15 ^a^	0.38 ± 0.07 ^a^	0.14 ± 0.04 ^a^
90% NI + CP-PBS	7671.15 ± 3028.31 ^ab^	0.79 ± 0.08 ^a^	0.28 ± 0.09 ^ab^	0.10 ± 0.02 ^ab^
CP-PBS	6274.01 ± 1387.86 ^b^	0.75 ± 0.10 ^a^	0.26 ± 0.03 ^b^	0.09 ± 0.01 ^b^

^a, b^ Mean values in the same row followed by different letters are significantly different (*p* < 0.05).

## Data Availability

The data that support the findings of this study are available from the corresponding author upon request.
